# Sustainable Nanotechnologies for Curative and Preventive Wood Deacidification Treatments: An Eco-Friendly and Innovative Approach

**DOI:** 10.3390/nano10091744

**Published:** 2020-09-03

**Authors:** Giuliana Taglieri, Valeria Daniele, Ludovico Macera, Ralf Schweins, Sandro Zorzi, Marie Capron, Gilles Chaumat, Claudia Mondelli

**Affiliations:** 1 Department of Industrial and Information Engineering and Economics, University of L’Aquila, Monteluco di Roio, 67100 L’Aquila, Italy; giuliana.taglieri@univaq.it (G.T.); valeria.daniele@univaq.it (V.D.); ludovico.macera@graduate.univaq.it (L.M.); 2 Institut Laue Langevin, 71 Avenue des Martyrs, CEDEX 9, 38042 Grenoble, France; schweins@ill.eu; 3 Department of Molecular Sciences and Nanosystems, Università Ca’ Foscari Venezia, via Torino, 155/b-Edificio ETA, 30172 Venezia-Mestre, Italy; 849103@stud.unive.it; 4 European Synchrotron Radiation Facilities (ESRF), 71 Avenue des Martyrs, CEDEX 9, 38042 Grenoble, France; marie.capron@esrf.fr; 5 Partnership for Soft Condensed Matter PSCM, 71 Avenue des Martyrs, CEDEX 9, 38042 Grenoble, France; 6 ARC-Nucleart CEA, 17 Avenue des Martyrs, CEDEX 9, 38054 Grenoble, France; gilles.chaumat@cea.fr; 7 CNR-IOM-OGG, Institut Laue Langevin, 71 Avenue des Martyrs, CEDEX 9, 38042 Grenoble, France

**Keywords:** wooden samples deacidification, eco-friendly treatments, Mg(OH)_2_ nanoparticles, Ca(OH)_2_ nanoparticles, SANS

## Abstract

Waterlogged wooden artifacts represent an important historical legacy of our past. They are very fragile, especially due to the severe phenomenon of acidification that may occur in the presence of acid precursors. To date, a satisfactory solution for the deacidification of ancient wood on a large scale has still not been found. In this paper, we propose, for the first time, eco-friendly curative and preventive treatments using nanoparticles (NPs) of earth alkaline hydroxides dispersed in water and produced on a large scale. We present the characterization of the NPs (by X-ray diffraction, atomic-force and electron microscopy, and small-angle neutron scattering), together with the study of the deacidification efficiency of our treatments. We demonstrate that all our treatments are very effective for both curative and preventive aims, able to assure an almost neutral or slightly alkaline pH of the treated woods. Furthermore, the use of water as a solvent paves the way for large-scale and eco-friendly applications which avoid substances that are harmful for the environment and for human health.

## 1. Introduction

In the field of preservation of the cultural heritage of humanity, the conservation of waterlogged wooden artifacts is extremely important since these artifacts represent an informative but extremely rare source of knowledge. Indeed, wooden relics contain important evidence about the main techniques and raw materials used for structures and artefacts, providing unique information about the economy, industry and buildings of ancient human societies. In addition, waterlogged environments usually provide the best possible conditions for limiting the decomposition of wood over time, as can be seen from the well-preserved archaeological objects and ships found after many centuries under the sea or at the bottom of lakes [1]. Nevertheless, the biological, chemical and mechanical changes caused by acid hydrolysis during long-term exposure to a marine or underground environment make the preservation of wooden artifacts difficult [2]. On the one hand, microbial organisms (in particular, erosion bacteria, but also soft rot fungi and scavenging bacteria) slowly attack waterlogged woods, even under near anoxic conditions, mainly damaging the cellulose and hemicellulose [3,4,5,6,7,8]. Moreover, the iron present in the wooden structures (for example, due to the presence of nails) corrodes and accelerates the degradation of the cell components. Anaerobic bacteria also contribute to the presence of iron sulfides within wooden structures, (e.g., pyrite and pyrrhotite) by breaking down the abundant iron minerals present in the soil and by using sulfur from decaying vegetation [9]. If the reduced sulfur species cause little threat in anoxic conditions, once exposed to oxygen some of them can easily oxidize and form sulfuric acid. This causes an irreversible and visible acid degradation of the wood via the loss of cellulose.

Due to the fragile conditions of waterlogged woods, at first, research into treatments for waterlogged wood was mainly aimed at developing methods to prevent damage due to stress during the drying process. The most common treatment of waterlogged wood involved using an aqueous solution of polyethylene glycol (PEG) to replace the water and prevent cracking while the wood was drying [10]. A mixture of PEGs of different molecular weights was most often the best solution for reducing dimensional changes during the drying process [11,12,13,14,15,16,17,18]. Nevertheless, all too often, these conventional procedures, which were designed to protect and save the wood samples, actually failed due to the formation, after many years from the treatment, of visible precipitates of acidic salts causing a pH ≤ 3.5 on wood surface. These phenomena could be related both to the presence of iron, which is active in the degradation of the end groups of poly (ethylene glycol), giving formic acid [19,20], and to the ion-conducting property of solid PEG as well. With the introduction of nanotechnology in cultural heritage research, attention was focused on the use of alcoholic dispersions of calcium hydroxide (CH) or magnesium hydroxide (MH) nanoparticles (NPs). These treatments seemed able, in a single-step, to stop the degradation processes due to metal ions and acidity and at the same time provide an alkaline reserve inside the wood [12,21,22,23,24]. However, despite the promising results obtained on small samples, they could not be used in large-scale treatments (conservation of boats, bridges and large artifacts), due to the lengthy and costly production routes involved [25]. For these reasons, it was proposed the use of strontium carbonate (SrCO_3_) NPs, obtained after milling the initial commercial powders using a dedicated procedure, and then dispersed in 2-propanol. These NPs represented a potential solution for both the immediate and long-term threats of acid degradation, as they were able to react with acidic sulfates to form insoluble SrSO_4_ precipitates. However, the SrCO_3_ NP dispersions can determine limitations for large-scale treatments as well, due to the necessity to maintain under sonication the wooden samples in the alcoholic dispersion for several hours [25]. Therefore, to date, a satisfactory solution to the problem of ancient wood deacidification for use on a large-scale has still not been found, limiting the prospects for effectively saving a large portion of historically relevant artifacts.

In the present paper, we consider the reassessment of colloidal suspensions of CH and MH NPs for deacidification treatments of archeological wood samples, containing the acidifying precursors (iron and sulfur), from a Gallo-Roman shipwreck loaned to the Atelier de Recherche et de Conservation ARC-Nucléart (Grenoble, France). In addition to the archeological and historical importance of the relict itself, the originality of this research relates to the fact that both CH and MH NPs to be used for the deacidification treatments are produced by means of an innovative and scalable synthetic procedure [26,27,28], which can give the possibility to extend the treatments not only on small samples but also on an extensive scale. Indeed, this procedure represents a versatile, sustainable and cost-effective synthetic route based on an ion-exchange process which occurs in water, at room temperature and ambient pressure, and operating with cheap or renewable reactants. Furthermore, in a single-step lasting 15 min, the process allows producing pure and crystalline alkaline earth hydroxide NPs without any intermediate steps to eliminate secondary products or additives, thus drastically reducing the overall synthesis times and assuring very high yields of production (>90%) [29,30,31,32,33,34]. All these factors offer the possibility for a scalable and sustainable NPs production which can provide large volumes of NPs dispersion for large-scale deacidification treatment. In addition, the strength of this method of synthesis is also based on the fact that the NPs are directly produced in water, leaving open the option of using them either in aqueous or in alcoholic dispersion. In this respect, we tested the deacidification efficiency of the alcoholic suspensions to compare our results with those reported in literature and, in parallel, we investigate for the first time the possibility to perform the deacidifying treatments using the MH NPs directly in aqueous suspensions. The use of water as a solvent is of paramount importance and represents a real breakthrough in this field, making it possible to fulfill the requirements of large-scale applications and promote the use of low-cost, safe and eco-friendly products. Alcoholic solvents actually increase the costs and they are also flammable and volatile organic compounds (VOCs), therefore posing serious risks if employed on large volumes. More stringent Health and Safety regulations have established that “VOC emissions produced by alcohol are harmful both to the environment and to human health as well as posing a fire hazard in the early stages” [35].

In addition, the present study focuses on the use of alkaline-earth hydroxide NPs, produced by the sustainable and scalable process, both for curative and, in an original way, also for preventive treatments of the waterlogged wood samples containing acid precursors. Indeed, if curative treatments have already been reported in literature, to our knowledge there has been very little written about preventive treatments. Specifically, for the preventive aim, some samples were treated with the NPs suspensions before an acidification process had begun, while, for the curative treatments, acidic or properly acidified samples were used. Preventive treatments include all direct measures and actions aimed at avoiding and minimizing future deteriorations or loss of materials or artefact, while curative treatments includes all actions directly applied to an object aimed at arresting damaging processes and, when possible, stabilizing their conditions against further deterioration. Moreover, for the first time, we propose the use of pure aqueous stable dispersions of MH NPs, in order to provide an eco-friendly approach, from synthesis to application, which could be of paramount importance for extensive treatments.

In order to investigate main features of the wood samples, to analyze the structural and morphological features of the new synthesized CH and MH NPs, and to study the efficiency of the deacidifying treatments, we used several non-destructive methods, such as optical microscopy (OM), scanning electron microscopy (SEM) equipped with an X-ray probe (SEM-EDX), Fourier transform infrared spectroscopy (FTIR), X-ray powder diffraction (XRD), Transmission Electron Microscopy (TEM), Atomic Force Microscopy (AFM), Small-Angle Neutron Scattering (SANS) and pH measurements.

## 2. Materials and Methods

### 2.1. The Wood Samples from the “Lyon Saint-George 4”, Gallo-Roman Wreck

**Figure 1:**
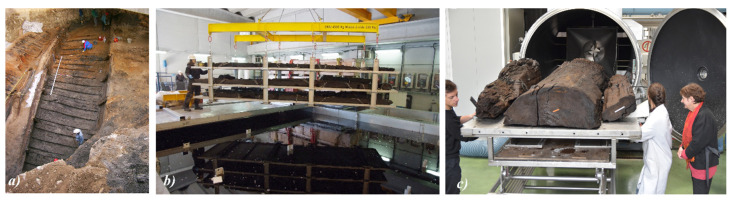
(**a**) Excavation site in Lyon; (**b**) the large pools containing the polyethylene glycol (PEG) where the wreck is immersed to prevent the structure from collapsing and cracking during wood drying; (**c**) drying process carried out inside the apparatus for large-scale lyophilization procedure.

The samples used in our work come from a wooden Gallo-Roman wreck (dated back to second century BC), discovered in Lyon (France) in 2003 by archeologists from the INRAP (French Institute for Archeological Research and Conservation) during the excavation work near the river Saône (Figure 1a). The wreck, baptized the Lyon Saint-Georges 4 (LSG4), was about 17 m long and was cut into 6 parts and stored in a lake near Lyon until 2014, when the French authorities decided to take it out of water and begin recovery and conservation treatments.

**Figure 2:**
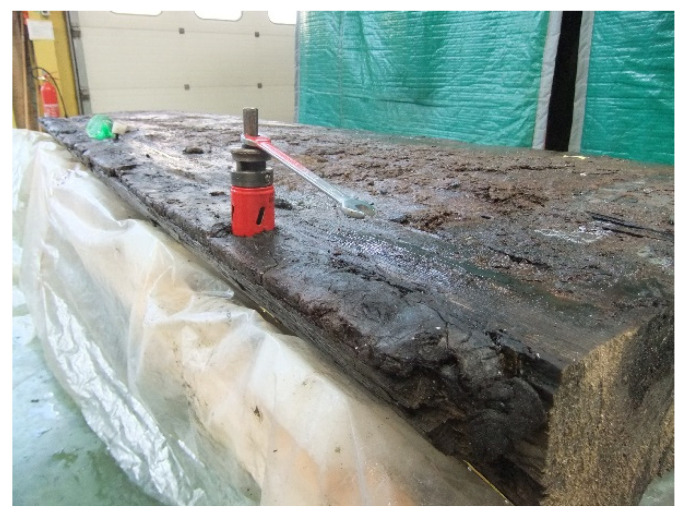
Sampling the core to evaluate the concentration of the acid precursors: coring drill around a nail position.

The workshop ARC Nucléart was assigned the conservation and restoration work. The first step involved removing the metal parts (nails, etc.), salt efflorescence, mud and debris (quartz and calcite) covering the surface of the wreck. The next step was to immerse the wreck in pools containing an aqueous solution of 20 wt.% of PEG 200 (Merck KGaA, Darmstadt, Germany), followed by a treatment with 35 wt.% of PEG 2000 (Merck KGaA, Darmstadt, Germany), for a minimum of 4 months respectively (Figure 1b), to prevent the structure of the wood from collapsing during the subsequent drying process. Moreover, in order to evaluate the concentration of iron and sulfur present at the nail/timber interface, after the removal of the nails, a core sample was made using a 35 mm hole saw around the hole left by the nail (Figure 2).

The assembly was frozen and then lyophilized. The core was impregnated with a polyester/styrene resin for two weeks and irradiated to harden the resin. In order to have a perfectly flat surface for SEM analysis, the sample was cut with a diamond saw along the radial axes, which were then carefully polished. A Philips XL 30 (F.E.I. Company, Hillsboro, OR, USA) scanning electron microscope equipped with an X-ray probe (SEM-EDX, F.E.I. Company, Hillsboro, OR, USA) was used to map sulfur and iron surrounding where the nails had been on the core samples. Then a few expendable samples, taken in different areas placed around iron nails and all containing the acid precursors, were used to test the effectiveness of our NPs both to prevent and to cure the acidification phenomenon in the wood, as will be described later. Specifically, the samples were only slightly acidic (referred as “non-acidic samples” in this paper). In addition, a single, particularly acidic sample (referred to as “acidic sample” in this paper) was available, and we used to test the curative treatment as well. Concerning the dimensions of the available samples, all contaminated by acid precursors, they are irregular and ranged approximately from (2 × 1 × 1) cm^3^ up to (5 × 3 × 2) cm^3^ similar to or slightly bigger than samples previously considered to test the efficacy of alcoholic alkaline nanoparticles suspensions [12]. We wish to stress that our choices for the experimental plan in the present study were driven by the availability of the samples for the different treatments. In particular, the shortage of historical acidic samples limited dramatically the number of analyses we could perform.

### 2.2. Synthesis and Characterization of CH and MH NPs

The new synthesized CH NPs were prepared by means of an ion exchange process, as described in the procedure reported in the European patent [26]. Concerning the synthesis of MH NPs, we followed a procedure similar to the one used for the CH NPs production, as previously reported [27]. From both the synthetic processes, we obtained CH and MH NPs dispersed in water with a suspension concentration of about 8 g/L. Moreover, by means of a laboratory scale reactor, the process allows producing, in a sustainable way, up to 400 g of NPs per day, corresponding to 50 L of NPs suspension, ready to be use for the deacidification treatments, without any washings or purification steps.

The structure, composition and crystallinity of the NPs were analyzed by X-ray Powder Diffraction (XRPD) pattern. The measurements were performed on the powders obtained by drying the suspension in an oven at 110 °C, taking care to avoid the reaction with atmospheric CO_2_. The experimental patterns were obtained by a PANalytical X’PertPRO diffractometer (CuKα radiation, Malvern Panalytical, Almelo, The Netherland) in the 10°–60° 2θ angular range, equipped with a solid-state detector (PIXcel, PANalytical, Malvern Panalytical, Almelo, The Netherland), in constant steps of 0.013° 2θ (time per step 1600 s). The patterns were then elaborated by means of a Profile Fit Software (HighScore Plus software package, PANalytical, Malvern Panalytical, Almelo, The Netherland), and crystalline phases were attributed by ICDD and ICSD reference databases. By analyzing the broadening of the diffraction peaks, we evaluated the corresponding crystallite size, D_hkl_, (i.e., coherent X-ray scattering domains) by using the Debye-Scherrer formula [36]. The morphology and size of the synthesized CH and MH NPs were investigated using both TEM (Philips CM100, F.E.I. Company, Hillsboro, OR, USA) and AFM (Cypher Asylum Research available at the AFM platform of the PSCM, in tapping mode, Oxford Instruments, High Wycombe, UK). The samples were prepared in accordance with the standard procedures, working under a nitrogen atmosphere in order to avoid any reaction with CO_2_. Furthermore, we performed SANS measurements to study the particles directly in suspension in their solvent. The SANS measurements were performed on the D11 instrument at the large-scale facility Institut Laue-Langevin, in Grenoble, France [37]. We used different configurations of the instrument in order to cover a large region in Q from 0.001 to 0.5 Å^−1^. We measured at a wavelength of 6 Å at three distances of the detector (1.2, 8.0, 28.0 m) and at a wavelength of 10 Å at 39.00 m. The measurements were performed in a standard Hellma cell made of quartz of 1 mm thick at room temperature. We used D_2_O as the solvent to reduce the incoherent signal coming from the hydrogen atoms of the solvent itself. The SANS curves I (Q) were obtained by integration of the normalized 2D intensity distribution with respect to the azimuthal angle after a standard data reduction taking into account the corrections for the empty cell contribution, transmission and detector efficiency using standard routines available at ILL. I (Q) curves were fitted with SasView software (this work benefited from the use of the SasView application, originally developed under NSF Award DMR-0520547).

### 2.3. Preventive and Curative Treatments on the Dry Wood Samples by Using CH and MH NPs

**Figure 3:**
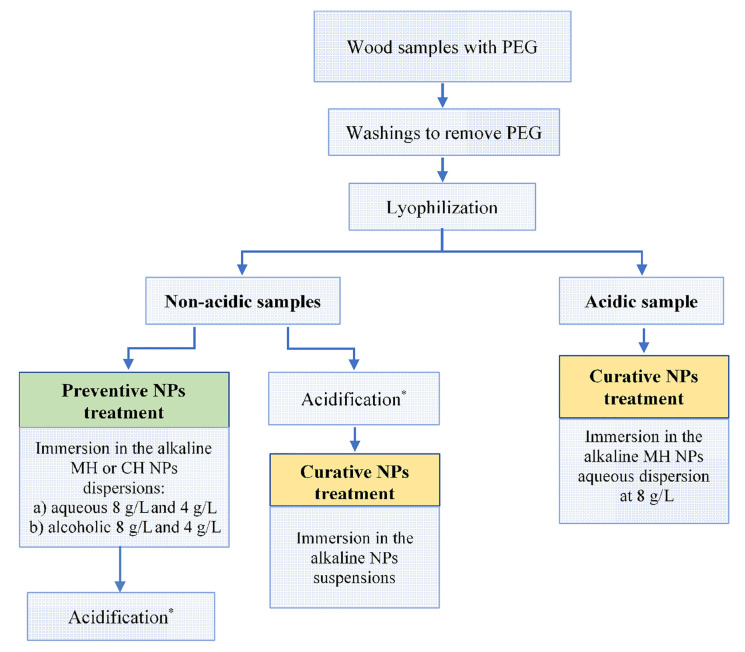
Scheme of the procedures followed for preventive and curative treatments of the Gallo-Roman wreck samples containing the acid precursors (iron and sulfur). * Acidification: hydrothermal ageing of the wood samples carried out at T = 80 ± 2 °C and relative humidity (RH) = 80% ± 5%, for 48 h.

We prepared the selected wood samples for NPs treatment, washing them in deionized water in order to remove the PEG, which could prevent the penetration of the NPs in the wood channels (vessels, water-conducting cells). To completely remove PEG form the treated wood, the samples were immersed in deionized water for several days and washed regularly until we obtained clear water. After the washing process, the samples were dried by means of a lyophilization procedure, which reduces the risk of damage or cracks, which generally result from drying in an oven or in air [38]. Once washed and dried, the samples were ready for the NPs treatments, whose main steps are schematized in Figure 3.

Specifically, we considered the acidic sample only for the curative treatment, while the non-acidic samples (but containing the acid precursor as well) were divided in two groups: the first group was earmarked for analyzing the curative NPs treatments, and the second group for the preventive NPs treatments. In all cases, the treatment was carried out by immersion of the wood samples in the NP suspensions for 30 days, considering an amount of nanoparticles dispersion over grams of wood of about 30 mL/g. In the case of the non-acidic samples, an acidification process was considered and it consisted in a hydrothermal ageing, carried out as follows: the samples were maintained at relatively high temperature (T = 80 ± 2 °C) and high relative humidity (RH = 80% ± 5%) for 48 h [12,22]. In particular, to evaluate the curative efficacy of the NPs treatment, the acidification was performed before the treatment; while, to evaluate the preventive efficacy the acidification was performed after the NPs treatment.

Concerning the MH-based treatments, we considered both alcoholic (2-propanol) and aqueous suspensions. We particularly used the aqueous MH suspension, which represents our new eco-friendly sustainable product, at a concentration of 8 g/L. This concentration was chosen because it is perfectly stable [27], thus guaranteeing a more homogeneous dispersion over long periods, which is necessary for the immersion of large objects. In order to compare these results with previous literature, we also prepared the alcoholic MH suspensions at two different concentrations: 4 g/L (to compare with literature results) and 8 g/L (to compare with our aqueous suspension), respectively. For the CH-based treatments, we used only alcoholic suspensions and we did not investigate the aqueous dispersion because of its low stability over time [29].

In summary, for the treatments of the non-acidic samples, we considered six suspensions, four based on the MH NPs dispersed either in alcohol (a), or in water (w), and two based on the CH NPs dispersed in alcohol. They are named as follows: MH_a4_, MH_a8_, MH_w4_, MH_w8_, CH_a4_ and CH_a8_, where the subscripts “8” and “4” refer to the concentration of 8 g/L and 4 g/L, respectively.

Finally, the suspension that showed the best deacidification efficiency was used for the curative treatment of the acidic sample (Figure 3).

### 2.4. Analysis of the Wooden Samples and of the Efficiency of the Treatments

The selected wood samples were analyzed using several techniques, before and after the washing process as well as after the NP treatments. The morphological features were investigated by optical stereomicroscopy (SM, Leica Stereozoom S8APO microscope, Leica Microsystems, Wetzlar, Germany) and SEM (SEM-BSE, Philips XL30CP, F.E.I. Company, Hillsboro, OR, USA). In addition, we used FTIR (Thermo Nicolet Nexus instrument, Thermo Fisher Scientific, MA, USA) to collect data on the molecular structure of the untreated wood samples, while X-ray Fluorescence (XRF, Philips PW2440, Panalytical, Almelo, The Netherlands) and XRPD were used to analyze the chemical and mineralogical composition of the acid precursors present inside the samples. XRD analyses were also used to evaluate the penetration of the NPs inside the wood structure, analyzing separately the surface and the core of treated samples. For XRF and XRD analyses, the samples were put in a desiccator for 24 h, finely grounded in an agate mortar, and then sieved and analyzed in the form of powders. Each experimental diffraction pattern was elaborated by a Profile Fit Software, and crystalline phases were attributed by ICDD and ICSD reference databases. Quantitative analyses by means of the Rietveld refinement method were also carried out. X-ray data were fitted using the pseudo-Voigt profile function. We refined the polynomial coefficient for the background function, lattice parameters, profile parameters, and Gaussian and Lorentzian profile coefficients [39]. Regarding the pH measurements, which are a crucial step in assessing the efficiency of the NPs treatments, we followed the procedure reported in the literature [22]: 1 g of wood powder was suspended in 5 g of deionized water for 24 h, in a closed container. Then, we measured the pH value of the supernatant water by means of a digital pH-meter, equipped with a combined electrode (Mettler-Toledo, model S220, Mettler Toledo, Columbus, OH, USA).

## 3. Results

**Figure 4:**
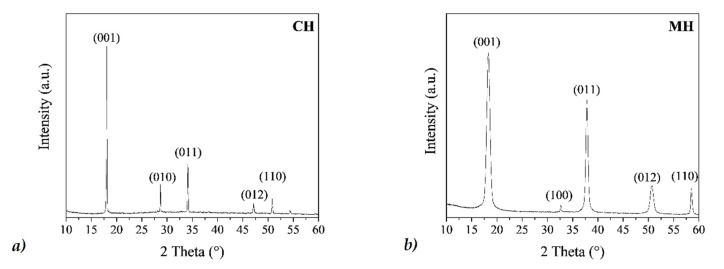
X-ray powder diffraction (XRD) patterns of (**a**) calcium hydroxide (CH), and (**b**) magnesium hydroxide (MH) dry powders. Bragg peaks are indexed by Miller notation.

From XRD investigations (Figure 4), performed on the dry powder obtained from the MH and CH aqueous suspensions, the patterns perfectly matched the hexagonal brucite structure (ICSD #98-016-9979) and the hexagonal portlandite structure (ICSD #98-020-2220), respectively, with no secondary phases, therefore denoting the formation of pure crystalline Mg(OH)_2_ or Ca(OH)_2_ compounds. We observed that the CH and MH dry powders showed a different broadening of the Bragg peaks, denoting larger crystalline domains in the CH NPs than in the MH NPs. In particular, we obtained average <D> values of about (110 ± 5) nm for CH and (20 ± 1) nm for MH. 

**Figure 5:**
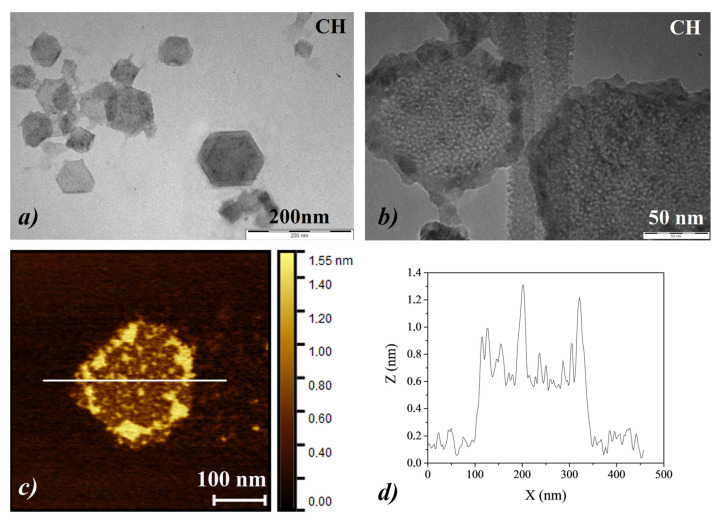
(**a**,**b**) Transmission Electron Microscopy (TEM) images of CH nanoparticles, acquired at different magnifications; (**c**,**d**) Atomic Force Microscopy (AFM) image of CH nanoparticles together with the corresponding profile analysis along the *Z*-axis of the primary nanoparticles (NPs).

From TEM and AFM characterizations, the CH NPs used for the treatments appear to be made up of pseudo-hexagonal lamellas, with side dimensions less than 100 nm (Figure 5a). Observed at higher magnification, each lamella appeared to comprise a self-assembly of primary nanoparticles with a size of less than 10 nm (Figure 5b) and a thickness of less than 2 nm, as shown by the AFM profiles along the *Z*-axis (Figure 5c,d).

**Figure 6:**
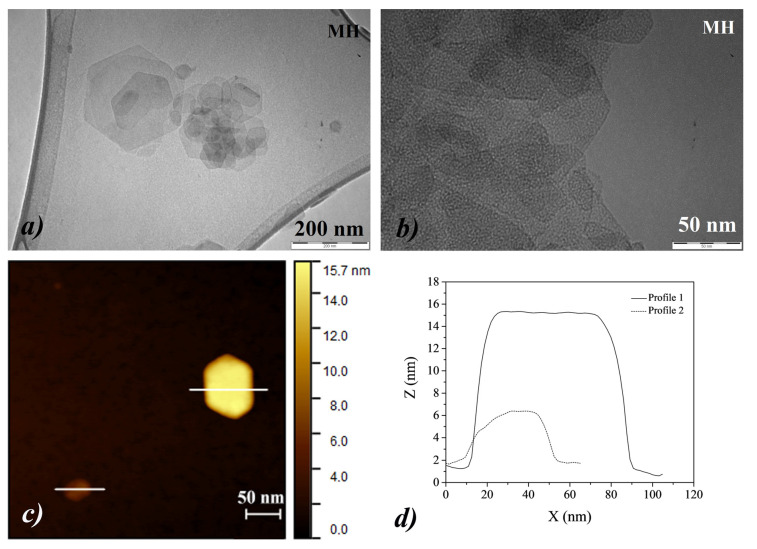
(**a**,**b**) TEM images of MH nanoparticles, acquired at different magnifications; (**c**,**d**) AFM image of MH nanoparticles together with the corresponding profile analysis along the *Z*-axis.

Similarly, the MH NPs appeared as hexagonal lamellas, with side dimensions generally less than 200 nm (Figure 6a). As observed for the CH NPs, at higher magnifications, each lamella appeared to consist of a dense superimposition of NPs, (Figure 6b,c), of few nanometers in size, as also previously investigated [27]. Finally, the observations by AFM revealed that the lamellas were characterized by a thickness generally ranging between 15 and 4 nm, (Figure 6d).

The size of the CH NPs obtained by microscopy seems to be inconsistent with the previous coherent X-ray lengths obtained by XRD. However, there is an easy explanation for this considering that during the procedure to dry the suspensions, the Ca(OH)_2_ colloidal particles tend to aggregate to form an epitaxial attachment [34,40], giving rise to a larger crystalline domain.

**Figure 7:**
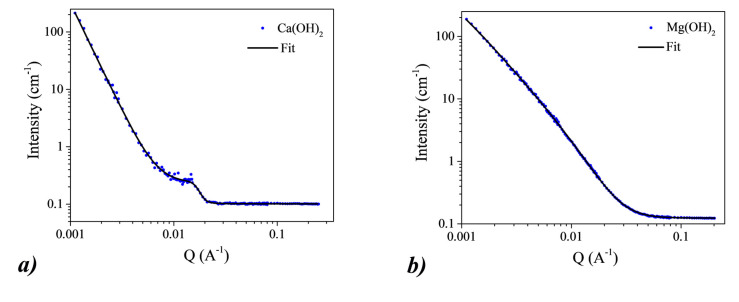
Small-angle neutron scattering (SANS) results on CH (**a**) and MH (**b**) nanoparticles. The experimental error bars may not be visible because they are smaller than the symbols for the experimental data. The fitting curves are represented by continuous lines.

The small-angle neutron scattering (SANS) measurements allowed us to study the form factor and dimension of the NPs directly in suspension. I (Q) curves are reported in Figure 7 with the corresponding fitting analysis. In the case of CH NPs, the best fit was obtained by using a correlation length model [41]. The model gives us a correlation length of 5.78 ± 0.02 nm, in agreement with the presence of primary particles of a size of less than 10 nm, as observed by AFM measurements.

Concerning the SANS data for the MH nanoparticles, the best fit was obtained by using a mass-surface fractal model with a modified Ornstein–Zernicke equation [42,43]. This model indicates the presence of aggregates formed by primary particles. In this specific case, the radius of gyration of the cluster r_g_ is 420 ± 40 nm and the radius of gyration of the primary particles r_g_ is 10.4 ± 0.2 nm. These results are completely consistent with what we obtained from the AFM measurements but give us the values of the size of the NPs in suspension rather than when dry. The different behavior of CH and MH NPs in suspension could reasonably be due to the fact that the two hydroxides interact differently with the solvent.

### 3.1. Study of the Wood Samples before Treatments

**Figure 8:**
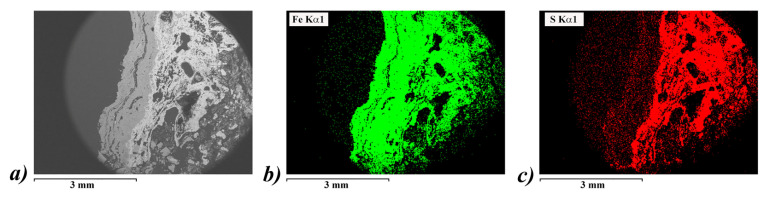
SEM images (**a**) of a representative polished section of a core around a nail together with the EDX mapping of the acid precursors iron (green, (**b**)) and sulfur (red, (**c**)). The EDX mapping is attributed to the signal of Kα emission.

A crucial step when studying the deacidification treatments on the wood samples is the characterization of the wood region around the position of the nail, that is at the origin of the presence of acidification precursors. We report, in Figure 8, the SEM image of a representative polished section of a core around a nail together with the EDX mapping of the acid precursors iron and sulfur. We can observe the diffusion of iron and sulfur along the radial section. As a result, iron can be found from the surface up to 4 mm thick (green zone) with higher concentration mainly localized along the inner surface. While sulfur compound is essentially localized in the external part up to about 1 mm from the inner surface.

**Figure 9:**
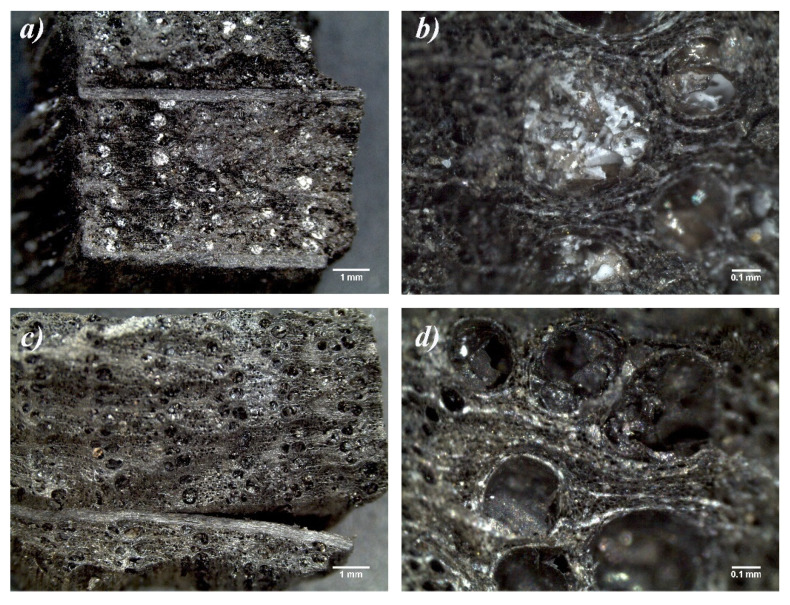
Optical stereomicroscopy images referred to the untreated dry wooden fragments before PEG washings (**a**,**b**) and after PEG washings (**c**,**d**).

The untreated dry fragments were analyzed by optical stereomicroscope in order to determine the nature of the wood samples (Figure 9). They present a cellular structure, typical of hard woods (oak). In fact, 35–70% of the wood volume is made up of fiber cells and 5–60% of the wood volume is made up of vessels [44], as shown in Figure 9a. Furthermore, the vessels are characterized by diameters ranging from 50 to 500 µm and they are organized in a semi-ring porous arrangement, as reported in the literature for hard woods [45]. The vessels were originally saturated with a whitish phase, due to PEG (Figure 9a,b) used in earlier treatments. This phase disappeared after the washing process (Figure 9c,d).

The molecular structure, analyzed by FTIR, was shown in Figure S1. We observed the presence of the typical absorption bands of lignin and hemicellulose, as confirmed by the comparison with the reference spectra of lignin, cellulose and hemicellulose [46,47]. No evidence of the typical absorption bands of cellulose was found. Indeed, in the case of buried woods, a relatively rapid removal of amorphous or paracrystalline cellulose occurs, and a slower conversion of crystalline to non-crystalline cellulose take place as well. For these reasons, only the residual lignin skeleton may be found in very ancient woods, which is responsible to maintain the shape of the object [48,49].

The XRF analyses on the untreated dry samples revealed the presence of Fe and S, confirming and quantifying the occurrence of acid precursors in the selected samples. Small amounts of Ca and Si were also measured, probably related to the impregnation of the wood for over 2000 years while the wreck remained underground and also in the lake. The corresponding XRD characterization showed mainly the presence of pyrite (FeS2, ICSD #98-005-3529); low percentages of quartz (SiO2, ICSD #98-0041672) and calcite (CaCO3, ICSD #98-001-8164) were also observed, in agreement with the XRF analysis. The quantitative results from the XRF and XRD measurements are summarized in Table 1.

### 3.2. Efficiency of the Treatments

We evaluated the deacidification efficiency of CH and MH NP suspensions by measuring the pH variations, as reported in Table 2. As described above, the treatments carried out on the “non-acidic” fragments were performed with both preventive and curative aims. We would like to stress that for untreated samples, the hydrothermal ageing caused a dramatic decrease in the pH value of about 70%, reducing from 5.31 ± 0.01 (slightly acidic) up to 3.70 ± 0.01 (definitely acidic). Indeed, the presence of the acid precursors during the hydrothermal ageing clearly accelerated the acidification process of the wooden samples, as expected due to the fact that high relative humidity is strongly linked to the development of acidity and due to the increased reactivity of the chemical processes with temperature [48]. It is a reliable estimate that the pH value reached after the hydrothermal ageing would be the final pH after a natural ageing, highlighting the necessity for preventive treatments, here proposed for the first time.

As can be seen in Table 2, all the preventive treatments proved to be highly effective, irrespective of the solvent concentration and the alkaline earth hydroxide used in the treatments themselves. This makes it possible to tailor the treatment to the specific requirements of the protective procedures being performed, something that is crucial if we consider the variety of needs that exist in conservation science. The results of our curative treatments on the artificially acidified samples were also highly satisfactory. In fact, all of them resulted in a neutral pH value in the treated samples. These results confirm the efficiency of alcohol-based treatments as already observed in the literature for similar suspensions [21,22,23]. However, we proposed a smarter option, paving the way for water-based curative treatments, representing an eco-friendly solution for large-scale applications, such as for wrecks and wooden artifacts. On the basis of these results and taking into account the fact that the aqueous suspensions of CH were not stable, we chose to use the MH aqueous suspension for the curative treatment of the very acidic sample (pH = 1.56 ± 0.01). After the curative treatment, even this extremely acidic sample was satisfactorily deacidified, as shown in Table 2. Another important aspect to be considered was the preservation of the aesthetic features of the treated samples, which must remain unchanged, from a visual observation, as far as possible. The observations indicated that the treatments, irrespective of the dispersing medium, did not altered the samples, and any white hazes or residual substance would be visible, by naked eyes, on the treated surfaces after the immersions in the NPs suspensions, as shown in Figure S2.

**Figure 10:**
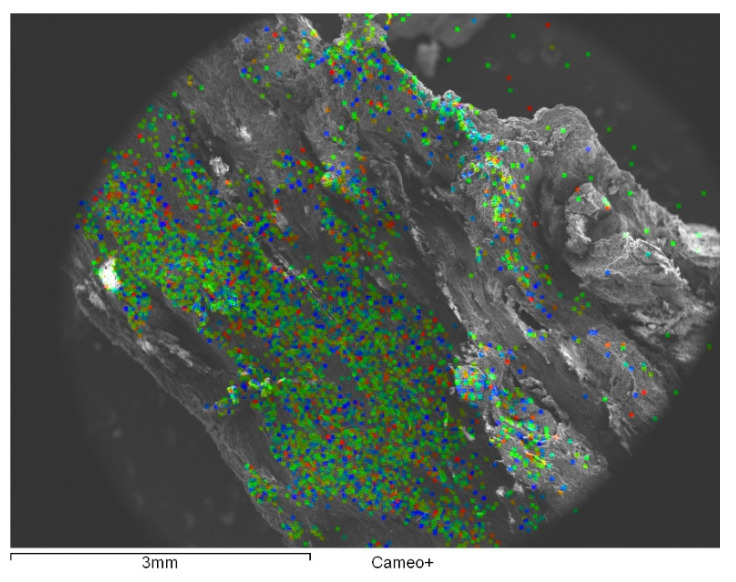
SEM-EDX image showed the penetrations of MH NPs inside the sample, following the Mg mapping.

An interesting parameter for conservation is the penetration of the treatments into the volume of the samples. We focused this analysis on the MH-in water treatment, as the best candidate for eco-friendly large-scale applications. To perform this analysis, we cut the treated sample longitudinally to the fibers in order to obtain a radial section (where the fiber walls of the wood can be distinguished). The X-ray microanalysis, carried out by SEM-EDX, revealed the presence of Fe and S, and traces of Ca and Mg. The mapping of Mg confirmed a homogeneous distribution of MH NPs throughout the whole depth of the sample, as shown in Figure 10.

XRD measurements confirmed the presence of MH NPs inside and on the surface of the treated wood (Figure S3). Inside and on the surface of the fragments, we found 13% and 23% of Mg(OH)_2_, respectively. These percentages are relative to the total amount of crystalline phases in the sample (pyrite, FeS_2_, and quartz, SiO_2_).

The treatments based on MH NPs dispersed in water, therefore, proved to be the best and most efficient method of deacidification in both preventive and curative actions, serving to prevent the many phenomena that dramatically affect the life of ancient artifacts over the course of time.

## 4. Conclusions

Nanotechnologies represent the future in conservation science. In this work, we demonstrate the high effectiveness of CH and MH NPs produced in aqueous suspension for both preventive and curative treatments of ancient wood. The nanoparticles used in this study were produced using a sustainable, one-step procedure, characterized by a low environmental impact, a high production yield, cost efficiency, and easy scalability in the production for uses in large-scale applications. Although effective solutions based on alcoholic dispersions of earth alkaline hydroxide NPs have been already proposed in several fields of conservation (including for wooden artefacts), their use on large surfaces is limited due to their limited production, high costs and to the use of alcoholic solvents as well. Until now there has been a lack of solutions for very large wooden artifacts, such as boats, bridges, etc. In the present study, we propose a new preventive approach for deacidifying treatments and compare the use of scalable productions of MH and CH NPs for curative treatments on acidified samples coming from an historically relevant Gallo-Roman shipwreck. Both CH and MH NPs were produced directly in water, but the CH NPs were used in the treatments only dispersed in alcohol (2-propanol) because of their tendency to agglomerate in water. The MH NPs were used in both 2-propanol or aqueous solvents. We demonstrated that all the treatments are very effective for both curative and preventive actions: the pH of the treated acidified wood changed from acid to neutral or almost neutral. Furthermore, the treatments were colorless, which is a crucial feature in treatments aimed at archaeological artifacts. Moreover, it is important to point out that this paper presented, for the first time, a study of a treatment based on alkaline earth NPs, produced on a large scale, in aqueous suspensions. The large volumes of NPs dispersions and the use of water as solvent are vital for large-scale applications, since it allows operating with large wooden objects without the use of volatile organic compounds that are flammable and harmful for the environment and for human health. In today’s society, where sustainability and environmental protection are crucial issues, the use of eco-friendly, non-destructive treatments is of fundamental importance for conservation science. As a result, this study will open new perspectives for the preservation of ancient artifacts, for large-scale deacidification treatments of waterlogged woods.

## Figures and Tables

**Table 1:** X-ray Fluorescence (XRF) and XRD quantitative analyses of the untreated dry wood samples.

XRF				XRD		
Fe	S	Ca	Si	FeS_2_	SiO_2_	CaCO_3_
15%	5%	0.8%	0.2%	95.5%	4.0%	0.5%

**Table 2:** Results of pH values refer to the “non-acidic” untreated wood samples, before and after the acidification treatment, and treated wood samples, in relation to the preventive and the curative treatments; results of pH values of the highly acidic wood sample, untreated and treated with curative treatment.

“Non-Acidic” Wood Samples			Acidic Wood Sample	
**Untreated**	pH before acidification	pH after acidification	pH Untreated	pH Treated with MH_8w_
	5.31 ± 0.01	3.70 ± 0.01		
**Treated**	pH preventive treatment #	pH curative treatment §	1.56 ± 0.01	6.65 ± 0.01
CH_4a_	7.53 ± 0.01	6.64 ± 0.01		
CH_8a_	7.52 ± 0.01	6.72 ± 0.01		
MH_4a_	7.83 ± 0.01	7.66 ± 0.01		
MH_8a_	7.89 ± 0.01	7.67 ± 0.01		
MH_8w_	7.83 ± 0.01	7.66 ± 0.01		

# pH values of the samples treated and then acidified by hydrothermal ageing; § pH values of the samples acidified and then treated by hydrothermal ageing.

## Supplementary Materials

The following are available online at https://www.mdpi.com/2079-4991/10/9/1744/s1, Figure S1: FTIR spectrum of the untreated dry fragments of wood samples, Figure S2. Visual inspections of the wood fragments both as received (untreated) and treated by means of CH_a4_, MH_a4_ and MH_w8_ suspensions, Figure S3: XRD patterns referred to the core and to the surface of the wooden samples treated by means of MH NPs.
