# Supplementary material for: Sustainable Nanotechnologies for Curative and Preventive Wood Deacidification Treatments: An Eco-Friendly and Innovative Approach
Source: Nanomaterials (Basel). 2020 Sep 3;10(9):1744. doi: 10.3390/nano10091744 (PMC7557752; doi:10.3390/nano10091744)
Supplement: Supplementary file 1 [file nanomaterials-10-01744-s001.pdf]

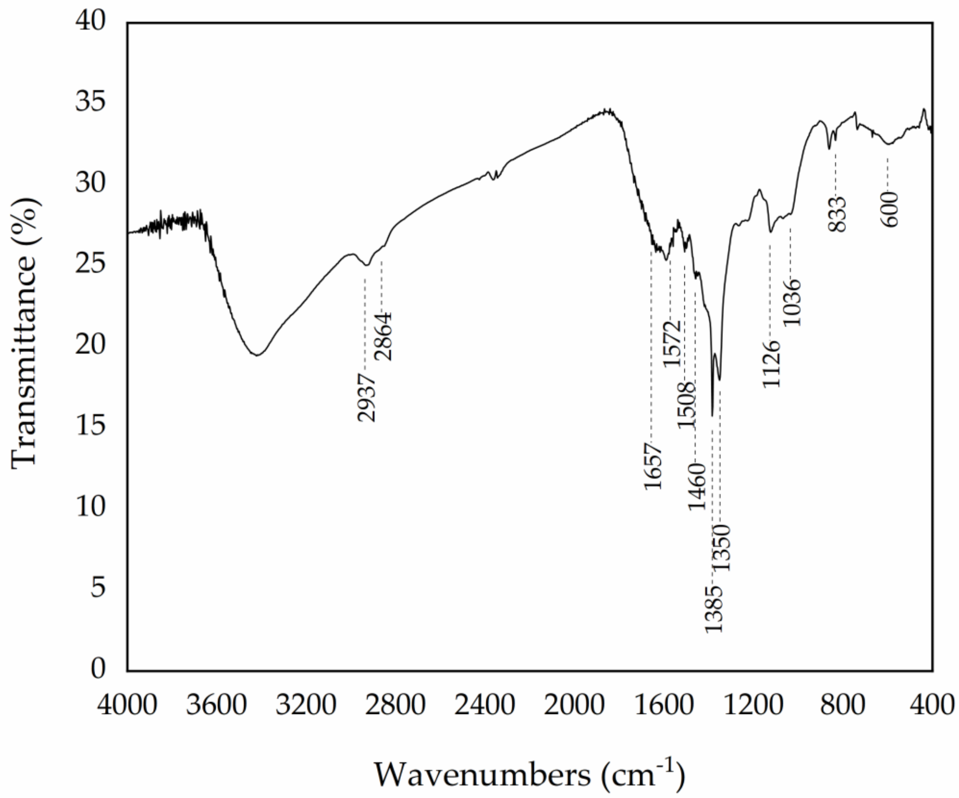

Figure S1. FTIR spectrum of the untreated dry fragments of wood samples.

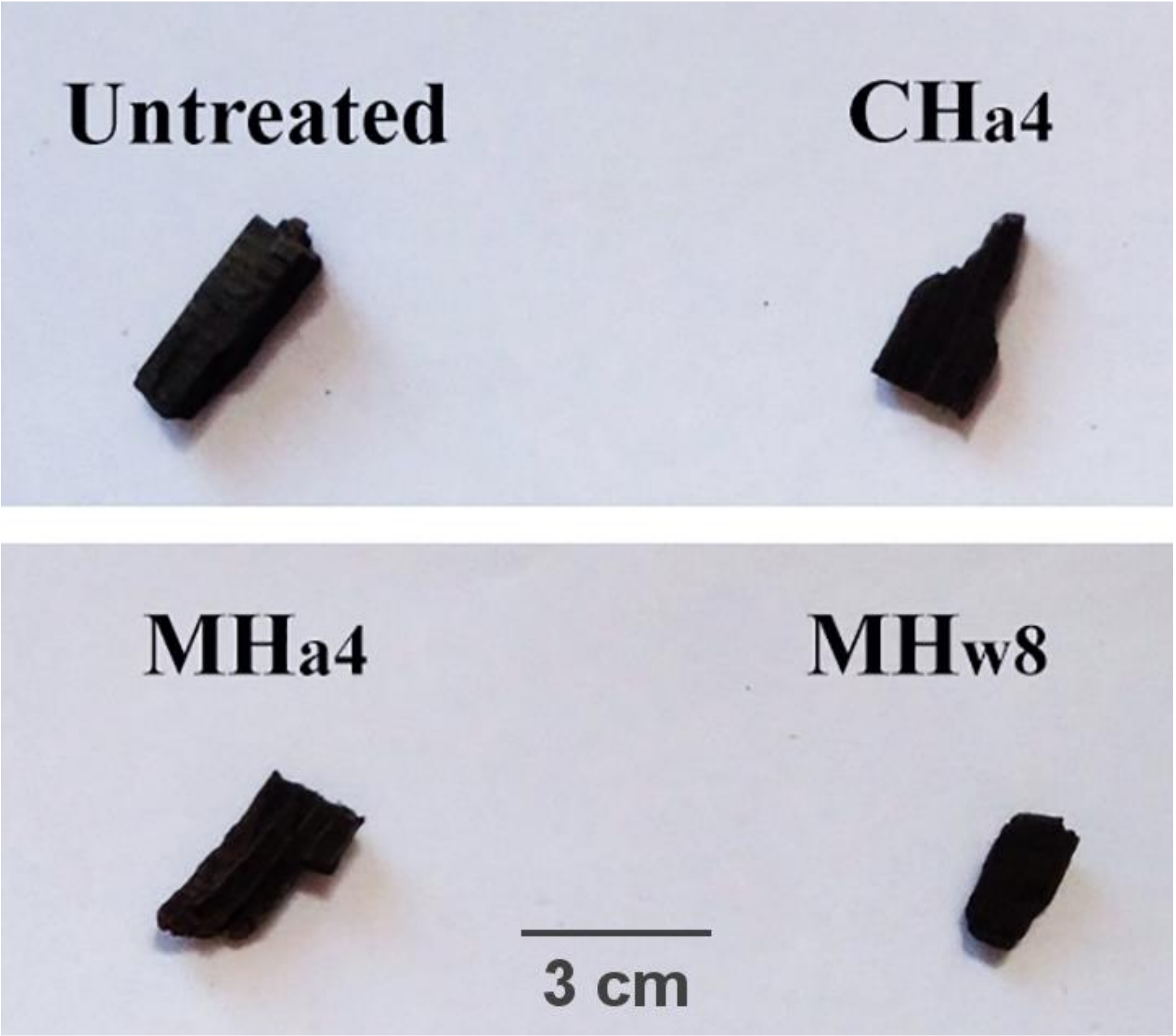

Figure S2. Visual inspections of the wood fragments both as received (untreated) and treated by means of CH_{a4}, MH_{a4} and MH_{w8} suspensions.

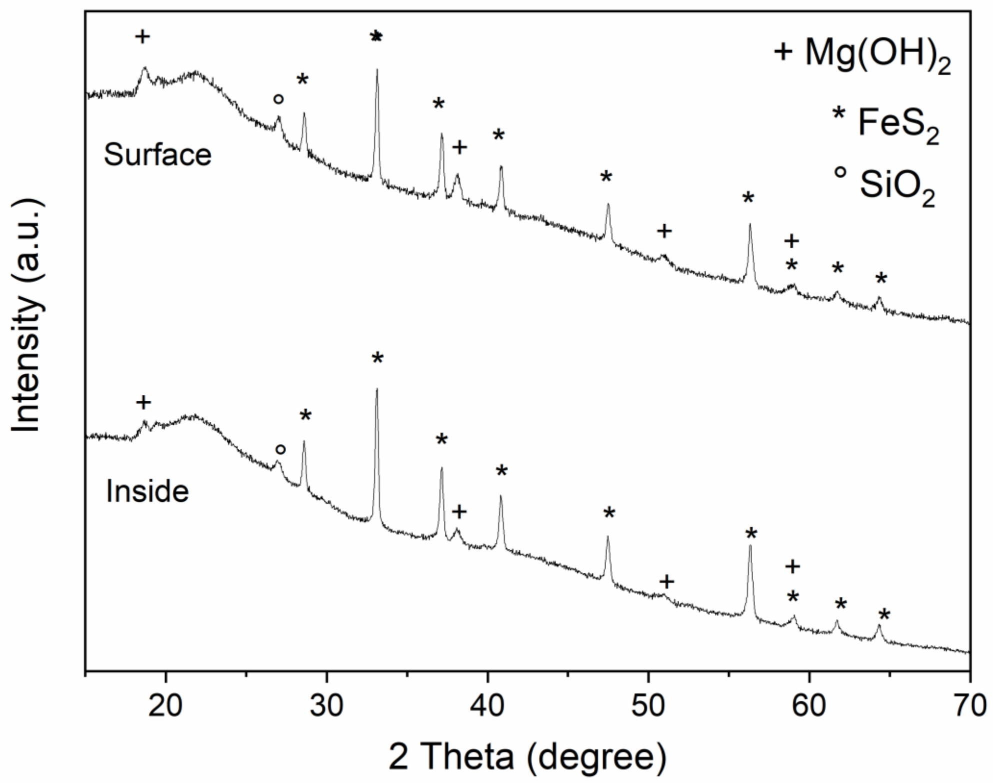

Figure S3. XRD patterns referred to the core and to the surface of the wooden samples treated by means of MH NPs.
